# Performance evaluation of different regression models: application in a breast cancer patient data

**DOI:** 10.1038/s41598-024-62627-6

**Published:** 2024-06-06

**Authors:** Mona Mahmoud Abo El Nasr, Alaa A. Abdelmegaly, Doaa A. Abdo

**Affiliations:** 1 Department of Applied Statistics and Insurance, Faculty of Commerce, Mansoura University, Mansoura, 33516 Egypt; 2 Higher Institute of Advanced Management Sciences and Computers, Al-Buhayrah, Egypt

**Keywords:** Cancer, Breast cancer

## Abstract

This paper provides a comprehensive analysis of linear regression models, focusing on addressing multicollinearity challenges in breast cancer patient data. Linear regression methodologies, including GAM, Beta, GAM Beta, Ridge, and Beta Ridge, are compared using two statistical criteria. The study, conducted with R software, showcases the Beta regression model’s exceptional performance, achieving a BIC of − 5520.416. Furthermore, the Ridge regression model demonstrates remarkable results with the best AIC at − 8002.647. The findings underscore the practical application of these models in real-world scenarios and emphasize the Beta regression model’s superior ability to handle multicollinearity challenges. The preference for AIC over BIC in Generalized Additive Models (GAMs) is rooted in the AIC’s calculation framework, highlighting its effectiveness in capturing the complexity and flexibility inherent in GAMs.

## Introduction

Regression analysis is one of the most important tools which has several applications in many fields. There are various types of regression models available in the literature, linear model (LRM), non-linear model, generalized linear model (GLM), and generalized additive models (GAM)^1^. GLM Introduced by Nelder & Wedderburn in 1972. GLM surpasses LRM assumptions, accommodating non-normally distributed responses, addressing heteroscedasticity, and allowing non-linear associations with predictors^2,3^. GLM takes many forms, one of these is the beta regression model (BRM), which models the continuous random variable dependency and suggests that the standard unit values are intervals based on the independent variables in different fields^4^. proposed BRM to explain variations in the dependent variable by rates and proportion behavior which supposes interval values (0, 1). This model assumes that the response variable follows the beta distribution. Further, the model can also accommodate asymmetries and heteroscedasticity^1^. Generally, the maximum likelihood estimator (MLE) is used to estimate the unknown regression coefficients of the BRM^5,6^. GAM offers the analyst an outstanding regression tool for understanding the quantitative structure of language data. An early monograph on generalized additive models is Hastie and Tibshirani in 1990^7^. GLM and GAM have become one of the standard tools for analyzing the impact of covariates on possibly non-Gaussian response variables. The only difference between GAM and GLM is that GAM permits the including nonlinear smooth functions in the model^8^. The selection of the smoothing parameter can be obtained, among many other proposals, by minimizing the conditional Akaike’s information criterion (AIC)^9^. This version of AIC for GAMs uses the log-likelihood evaluated at the penalized MLE and with the effective degrees of freedom computed as discussed in^10^.

Multicollinearity problem is a popular issue in regression modeling. It indicates that there is a strong association between the explanatory variables. However, many biased estimators have been introduced to combat multicollinearity in linear regression, such as the Stein estimator^11^, principal component estimator^12^, ridge regression estimator^13^, improved ridge estimators^14^, contraction estimator^15^, modified ridge regression estimator^16^, Liu estimator^17^, Liu-type estimator^18^, restricted and unrestricted two-parameter estimator^19^, (k-d) class estimator^20^, mixed ridge estimator^21^ and modified Liu-type estimator^22^. There are several methods to estimate the shrinkage parameter such as ridge, Liu, and Liu-type estimations, which have become a generally accepted and more effective methodology to solve the multicollinearity problem in several regression models^13^ proposed the ridge estimator (RE), the concept behind the ridge estimator is to apply a small definite amount (k) to the diagonal entries of the covariance matrix to increase the conditioning of this matrix, reduce the MSE, and achieve consistent coefficients. Several attempts have been made to choose the best ridge parameter k: Based on the work of^23^ and^24^. The impact of multicollinearity on GLM is significant and enduring. Among the various GLMs, the BRM is notably affected by multicollinearity^5,25^ and^26^ proposed the ridge estimators for the BRM to remedy the problem of instability of the traditional ML method and increase the efficiency of estimation^1^ proposed a new modified ridge-type estimator for the BRM. This paper aims to present a comparative analysis of various statistical models, incorporating both real data and simulation studies, with a specific focus on evaluating these models using the Akaike Information Criterion (AIC) and the Bayesian Information Criterion (BIC). Although there are a lot of high-dimension regression studies^27,28^, this paper specifically focuses on the evaluation of low-dimensional regression models. This paper is organized as follows; the differences in regression models the beta regression model, GAM regression model, GAM beta regression model, ridge model, and the beta ridge regression are presented. A numerical evaluation is offered using both Monte-Carlo simulation and empirical data application, respectively. Finally, conclusions are presented.

## Methodology

### Beta regression model

The most used model in many branches like economic and medical research is beta regression, which is used to consider the influence of specific independent variables on a non-normal dependent variable. However, in the state of beta regression, the response variable is constrained to intervals (0, 1), such as fractions and percentages. These models are used to examine the relationship and effect of some chosen independent and normal dependent variables. However, this is not appropriate for conditions where the response variable does not follow the normal distribution because it may give an overestimated estimator^4^ developed the beta regression model by using the link function to connect the mean function of its dependent variable and linear predictors. The inverse of a precision parameter of this model is called a dispersion scale, this parameter contains stability through observations. Despite the precision parameter might not be constant with the results of^29,30^.

Let *y* be a continuous random variable that has a beta distribution with a probability density function as follows:

1 $$\begin{aligned} f(y_{i},\mu ,\emptyset )&= \dfrac{\Gamma (\emptyset )}{\Gamma (\mu \emptyset ) \Gamma (1-\mu \emptyset )} y^{\mu \emptyset -1}(1-y)^{\emptyset -\mu \emptyset -1}, 0<y<1;\quad 0<\mu <1; \emptyset >0. \end{aligned}$$

where $\Gamma$ is the gamma function and $\emptyset$ is the precision parameter.

$$\begin{aligned} \emptyset =\dfrac{1-\sigma ^{2}}{\sigma ^{2}} \end{aligned}$$

The mean and variance of the beta probability distribution are:

$$\begin{aligned} E(y)&= \mu .\\ var(y)&= \mu (1-\mu )\sigma ^{2} \end{aligned}$$

By using the logit link function, the model allows $\mu _{i}$, depending on covariates as follows:

2 $$\begin{aligned} g(\mu _{i})&= \log \left( \dfrac{\mu _{i}}{1-\mu _{i}}\right) =X_{i} ^\prime \beta =\eta _{i} \end{aligned}$$

The linear predictor is constrained within the beta distribution, which inherently models data with in the open set (0, 1). In scenarios where extreme values at 0 and 1 are observable, one can consider employing the inflated zero and or one beta distribution proposed by^31^.

### Generalized Additive Model (GAM)

^32^ introduced generalized additive models that allow be modelling of the dependence of the response variable in a flexible way using smooth functions of the predictors by defining the linear predictors:

3 $$\begin{aligned} \eta _{i}&= \beta _{0}+\sum _{j=1}^{p}f_{j}(x_{ij}). \end{aligned}$$

where, $f_{j}(x_{ij})=\sum _{k=1}^{kj}\beta _{jk}(x_{ij})$ is the smoothing term from the $j^{\text {th}}$ predictor with $\{ \beta _{jk}( )\}_{k=1}^{kj}$, the asset of known basis functions associated with unknown parameter $\beta _{jk}$.

We can define different smoothers by adopting different basis functions.

As penalized regression splines, cubic regression splines bases^33^.

#### Estimation

We can estimate the GAM model using restricted maximum likelihood (REML), which amounts to maximizing the penalized log likelihood:

4 $$\begin{aligned} L_{p}(\beta )&= L(\beta )-\dfrac{1}{2}\lambda \beta ^\prime S\beta \end{aligned}$$

where $L(\beta )=\sum _{t=1}^{n}L(y_{i}/\beta )$ is the log-likelihood for the observed values $y_{i}$ of the response variable. $\lambda$: is a smoothing parameter. *S*: is a known penalty matrix. $\lambda \beta ^{T} S\beta$: the smoothing penalty.

Presented REML as a convenient estimation method for marginal likelihood estimation of $\beta$ when the model contains Gaussian random effects, and it also leads to more stable estimates of $\lambda$ with a much-reduced risk of under-smoothing^10,34^.

### GAM beta regression model

Let $y_i$ represent the test positive rate (TPR), which is determined by dividing the number of new positive cases $P_i$ by the total number of tests $T_i$ at time i. Time *i* is determined to have integer values between 1 and *n* for the first and last times of the period studied. TPR should have a built-in limit as a proportion between 0 and 1. Several methods and models may be used to analyze variables that are represented as proportions, but the beta regression model is perhaps the most well-known among them^5,35^.

The five-step GAM beta regression is as follows:

1. Suppose that the predictor variable $Y_i$ follows a beta distribution with a mean of $\mu _i$^14,16^.
  $$\begin{aligned} Y_i\sim \text {beta}(\mu _i,\emptyset ) \end{aligned}$$
  For the beta distribution’s mean and variance
  $$\begin{aligned} E(y_i)=\mu _i, \text {and } V(y_i)=\dfrac{\mu _i(1-\mu _i)}{1+\emptyset } \end{aligned}$$.
2. In the second stage, we define the model’s systematic component. We determine the linear predictive functional $\eta _i$ as:
  $$\begin{aligned} \eta _i=\beta 'x_i \end{aligned}$$
  where $\beta$ is a vector with a $(p+1)$ dimensional regression model parameters that are yet to be defined, and $x_i$ is the intercept plus the vector of measured values on *p* forecasters. The predictor function $\eta _i$ provides the systematic component^9^ and^36^. This equation represents how the systematic component is formulated in the model
3. In the third stage, we need to establish the relationship between the predictor function $\eta _i$ and the expected value of $Y_i$ denoted as $\mu _i$.. This relationship is achieved using the Link function, resulting in the following outcomes^9,36^.
  $$\begin{aligned} \mu _i=\dfrac{exp(\eta _i)}{1+exp(\eta _i)}=\dfrac{1}{1+exp(-\eta _i)} \end{aligned}$$
4. The Link function in Generalized Linear Models (GLM) is specified in the references^9,36^.
  $$\begin{aligned} logit(\mu _i)=\log \left( \dfrac{\mu _i}{1-\mu _i}\right) =\eta _i. \end{aligned}$$
5. Generalized additive models provide flexibility in modeling the dependence of the response variable by defining the linear predictor as a smooth function of the predictors, as described in^9^.
  $$\begin{aligned} \eta _i=\beta _0+\sum _{j=1}^{P}f_i(x_{ij}) \end{aligned}$$
  The term $f_i(x_{ij})=\sum _{k=1}^{kj}\beta _{jk}\beta _{jk}(x_{ij})$ represents the smoothing function for the $j^{\text {th}}$ predictor. It involves a sum of terms, each represented by $\beta _{jk}\beta _{jk}(x_{ij})$.
6. In estimating the Generalized Additive Model (GAM), Restricted Maximum Likelihood (REML) is utilized to maximize the penalized log-likelihood^9^ The penalized Log-Likelihood $L_p(\beta )$ is defined as
  $$\begin{aligned} L_p(\beta )=L(\beta )-\dfrac{1}{2}\lambda \beta ' S \beta \end{aligned}$$
  where $L(\beta )=\sum _{t=1}^{n}L(Y_i/\beta )$ is the likelihood function for the observed values $y_i$ of the response variable. $\lambda$ represents the smoothing parameter, and *S* is the known penalty matrix. The use of REML helps in maximizing this penalized log-likelihood for GAM estimation.
7. predictions can be calculated as^9^.
  5 $$\begin{aligned} \hat{\mu _{i}}&=\beta _{0}+\sum _{k=1}^{k} {\hat{\beta }}_{1k}\beta _{1k}(x_{i1}+(\hat{\beta _{2}}x_{i2})) \end{aligned}$$

### Ridge regression model

One of the most widely used techniques for solving multicollinearity in multiple linear regression is ridge analysis. This method has found applications in various fields, including engineering, chemistry, and econometrics. Ridge regression (RR) modifies the Ordinary Least Squares (OLS) method to produce biased estimators of regression coefficients, thereby addressing issues related to multicollinearity. This approach is particularly valuable when OLS estimators exhibit significant variability. So, ridge analysis can improve the predictability and accuracy of a model^13^. Here, we describe the linear regression model^37^:

6 $$\begin{aligned} Y&= X\beta +\epsilon \end{aligned}$$

where *Y* represents the dependent variable, it is an $n\times 1$, *X* is the matrix of predictor variables, $\beta$ is the vector of regression coefficients, it is $p\times 1$, and $\epsilon$ represents an $n\times 1$ vector of the error term.

In the context of ridge regression:

1. The ordinary least squares (OLS) estimator ${\hat{\beta }}$ Eq. (6) is calculated as follows
  7 $$\begin{aligned} {\hat{\beta }}&=(S)^{-1} X'Y \end{aligned}$$
  where $S=X'X$ is the design matrix. represents the design matrix, and ${\hat{\beta }}$ is the vector of regression coefficients estimated using the ordinary least squares method.
2. The ridge regression estimator, introduced by Hoerl and Kennard, is derived by minimizing the given objective function^37^
  8 $$\begin{aligned} (Y-X\beta )'(Y-X\beta )+k(\beta '\beta -c) \end{aligned}$$
  where $(Y-X\beta )'(Y-X\beta )+$ is a part of the OLS objective that minimizes the sum of squared residuals, and $k(\beta '\beta -c)$ is the penalty term, where *k* is a constant,$\beta$ is the vector of regression coefficients, and *c* is a predefined constant.
3. We obtain the normal equations^37^
  9 $$\begin{aligned} (X'X+KI_p)\beta&=X'Y \end{aligned}$$
  where $X'X$ is the sum of squares and cross-products matrix, $kI_p$ introduces the penalty term into the normal equations, and *k* is a constant.
4. The ridge estimator is determined by solving the normal equations, resulting in $({\hat{\beta }} (k))$ as shown in Eq. (10):
  10 $$\begin{aligned} {\hat{\beta }} (k)&= (S+kI_p)^{-1}X'Y=W(k){\hat{\beta }}. \end{aligned}$$
  where $S=X'X$, and $W(k)=(I_{P}+kS^{-1}) ^{-1}$ is a matrix derived to simplify the computation.
5. The parameter *k* is the Biasing Parameter in ridge regression, Eq. (11) provides a method for selecting it^13^.
  11 $$\begin{aligned} k&= p\sigma ^2/\beta '\beta . \end{aligned}$$
  where *p* is the overall output variable, $\sigma ^2$ is an estimate of the variance, and $\beta '\beta$ is the sum of squared estimated coefficients.
  The estimate of the ridge parameter, denoted as ${\hat{\beta }}_{k}$ is given by^38^:
  12 $$\begin{aligned} {\hat{\beta }}_{k}&= \Lambda +(kI)^{-1} X'Y \end{aligned}$$
  where $\Lambda$ represents a diagonal $P\times P$ matrix. Efficiency of ${\hat{\beta }}_k$ is influenced by the selection of the ridge parameter *k* to get the smallest Mean Squared Error (MSE) estimate, a certain *k* value is determined. This assessment is performed at a chosen value of *k*, as expressed in Eq. (13)^38^:
  13 $$\begin{aligned} \text {MSE}_{\text {RIGED} }&= \sum _{i=1}^{p}\dfrac{\lambda _i\sigma ^2+k\beta _{i}^2}{(\lambda _i+k)^2} \end{aligned}$$
  Here, unbiased OLS estimated values for ${\hat{\sigma }}^2$ and ${\hat{\beta }}$ are used in place of ${\sigma ^2}$ and ${\beta }$

### Beta ridge regression model

The beta ridge regression estimator is proposed as an alternative to the beta maximum. likelihood estimator to mitigate the impacts of multicollinearity in the Beta Regression model. This estimator is denoted as follows^5^ and^13^ .

Assuming that ${\hat{\beta }}$ is an estimator of the vector $\beta$, the weighted sum of squared error is defined as^5^:

14 $$\begin{aligned} \Theta&= (y-x\beta )'(y-x{\hat{\beta }})=(y-x{\hat{\beta }}_{\text {ML}})'(y-x{\hat{\beta }}_{\text {ML}}) ({\hat{\beta }}-{\hat{\beta }}_{\text {ML}})'X'WX({\hat{\beta }}-{\hat{\beta }}_{\text {ML}})\nonumber \\&=\Theta _{\text {min}}+\Theta {\hat{\beta }} \end{aligned}$$

where $\Theta$ represents the minimum value, and ${\hat{\beta }}>0$ is the constant increment that causes the WSSE to increase when ${\hat{\beta }}_{\text {ML}}$ substituted for ${\hat{\beta }}$. The BRR estimator is obtained by minimizing the Length of ${\hat{\beta }}$ subject to a restriction:

$({\hat{\beta }}-{\hat{\beta }}_{\text {ML}})^\prime X^\prime WX({\hat{\beta }}-{\hat{\beta }}_{\text {ML}})=\Theta _{0}$, as Hoerl and Kennard’s restrictions^13^.

Minimized $\varrho ={(y-{\hat{\beta }})\ }^\prime (y-{\hat{\beta }}) (\ y -{X{\hat{\beta }}}_{ML})\ ^\prime (y -{X{\hat{\beta }}}_{ML})+ (({\hat{\beta }}-{{\hat{\beta }}}_{ML})\ ^\prime X^\prime$ as Hoerl ${{\hat{\beta }}}_{ML}$

15 $$\begin{aligned} \varrho ={{\hat{\beta }}\ }^\prime {\hat{\beta }}+(1/k) (({\hat{\beta }}-{{\hat{\beta }}}_{ML}) \ ^\prime \ X^\prime W\ X ({\hat{\beta }}-{{\hat{\beta }}}_{ML})-\ \Theta _o) \end{aligned}$$

where the Lagrangian multiplier is 1/*k*. When Eq. (15) is differentiated from ${\hat{\beta }}$, the outcome equals zero.

$$\begin{aligned} \frac{\partial \varrho }{\partial {\hat{\beta }}}&=2{\hat{\beta }}+\frac{(2X^\prime W X({\hat{\beta }}-{{\hat{\beta }}}_{ML})}{k}=0. \end{aligned}$$

After simplification, we obtain the following BRR estimator:

16 $$\begin{aligned} {{\hat{\beta }}}_{BRR}&={\hat{\beta }}=(X^\prime WX+k{I)}^{-1}X^{\ ^\prime }W X{{\hat{\beta }}}_{ML} \end{aligned}$$

Where, *I* is a matrix of identities with an order of $p\times p$, and *k* is the shrinkage parameter.

## Numerical analysis

This study relies on data extracted from the Breast Cancer Wisconsin Diagnostic dataset, obtained from the University of Wisconsin Hospitals Madison Breast Cancer Database^39^, covering the period from January 1989 to November 1991. The dataset comprises records from 569 breast cancer patients and was accessed through an open online repository hosted at https://www.kaggle.com/code/gpreda/breast-cancer-prediction-from-cytopathology-data. Our research aims to explore the relationship between 10 predictor variables and tumor progression in breast cancer patients.

### Data set description

Breast cancer represents a significant health burden globally, standing as the most prevalent cancer among women and ranking as the second leading cause of cancer-related mortality in women. Characterized by aberrant cell growth in breast tissue, this disease poses substantial health risks. In our study, we selected the radius mean as the dependent variable for several reasons. While previous research predominantly focused on diagnosis and disease classification, our approach provides a novel perspective. By utilizing the diagnosis state to assess the extent of disease spread, as indicated by the radius mean variable, we delve into the progression of breast cancer based on diagnostic information. This unique method yields valuable insights into tumor behavior and disease severity. Utilizing ‘radius mean’ as a continuous variable enriches the analysis of tumor data, enabling the use of diverse statistical methods to uncover intricate patterns. This approach not only enhances the understanding of tumor impact on patient outcomes but also facilitates the discovery of new correlations and insights in breast cancer research^40^. The radius mean serves as the primary outcome variable in our analysis, representing the average distance from the cell center to the perimeter. Its importance lies in its association with tumor spread; as the cell radius increases, so does the surface area, indicating a more extensive tumor spread. Our investigation encompasses 10 predictor variables, including diagnosis, texture, perimeter, area, smoothness, compactness, concavity, concave points, symmetry, and fractal dimension. These variables play crucial roles in elucidating various aspects of breast cancer progression. Detailed units of measurement for the features in the Breast Cancer Wisconsin (Diagnostic) Data Set are provided in Table 1.

**Table 1:** Descriptive of variables.

Variables	Description
Radius	mean Mean of distances from the center to points on the perimeter (measured in pixels)
Diagnosis	Indicates two types: malignant (M) and benign (B). Malignant represents cancer
	Spreading to other parts of the body, while benign indicates non-cancerous growth
	With potential hormone therapy benefits and less aggressive treatment approaches.
Texture	The standard deviation of gray-scale values (measured in pixels).
Perimeter	Represents the mean value for the core tumor.
Area	Area of the core tumor (measured in square pixels).
Smoothness	Local variation in radius lengths.
Compactness	Perimeter2 /area 1.
Concavity	Severity of concave portions of the contour.
Concave Points	Number of concave portions of the contour.
Symmetry	Refers to the breasts that have different densities.
Fractal Dimension	Coastline approximation−1.

**Table 2:** Descriptive statistics of breast cancer variables.

Variables	N	Minimum	Maximum	Mean	Std. deviation
Texture	569	9.71	39.28	19.2896	4.30104
Perimeter	569	43.79	188.50	91.9690	24.29898
Area	569	143.50	2501.00	654.8891	351.91413
Smoothness	569	0.05	0.16	0.0964	0.01406
Compactness	569	0.02	0.35	0.1043	0.05281
Concavity	569	0.00	0.43	0.0888	0.07972
Concave points	569	0.00	0.20	0.0489	0.03880
Symmetry	569	0.11	0.30	0.1812	0.02741
Fractal Di1ension	569	0.05	0.10	0.0628	0.00706
Valid N (listwise)	569				

Table 2 provides comprehensive descriptive statistics for the variables in the breast cancer dataset, including the number of observations (N), as well as the minimum, maximum, mean, and standard deviation for each feature. Here’s a refined explanation of the analysis:

Texture, Perimeter, and Area: The mean texture value is 19.2896 (ranging from 9.71 to 39.28), the mean perimeter is 91.9690 pixels (ranging from 43.79 to 188.50), and the mean area is 654.8891 square pixels (ranging from 143.50 to 2501.00). Higher values for texture, perimeter, and area suggest greater variability, larger tumor sizes, and potentially more irregular tumor shapes, indicative of advanced breast cancer stages.

Smoothness and Compactness: The mean smoothness value is 0.0964 (ranging from 0.05 to 0.16), and the mean compactness is 0.1043 (ranging from 0.02 to 0.35). Lower smoothness values and higher compactness values suggest irregular and denser tumor structures, respectively, which may indicate more aggressive tumor growth patterns.

Concavity and concave points: The mean concavity value is 0.0888 (ranging from 0.00 to 0.43), and the mean number of concave points is 0.0489 (ranging from 0.00 to 0.20). Higher values for concavity and concave points indicate deeper and more pronounced concave regions in tumor contours, potentially reflecting aggressive tumor behavior.

Symmetry and fractal dimension: The mean symmetry value is 0.1812 (ranging from 0.11 to 0.30), and the mean fractal dimension is 0.0628 (ranging from 0.05 to 0.10). Deviations from symmetry in breast density and higher fractal dimension values suggest irregular and complex tumor shapes, respectively, which may be associated with aggressive tumor phenotypes and disease progression.

**Table 3:** Diagnosis.

		Frequency	Percent	Valid percent	Cumulative percent
Valid	B	357	62.7	62.7	62.7
	M	212	37.3	37.3	100.0
	Total	569	100.0	100.0	

In Table 3, the diagnosis frequencies indicate that 62.7% of cases are benign (B), while 37.3% are malignant (M). Understanding the distribution of malignant and benign cases is crucial for characterizing the dataset and identifying potential associations between diagnostic categories and clinical outcomes.

**Table 4:** The estimation of linear regression model parameters.

Variable	Estimate	Std. error	t	p. value
Intercept	$1.99 e^{-3}$	$1.35e^{-03}$	− 1.471	0.141901
Diagnosis	$4.97e^{-04}$	$1.33e^{-04}$	3.734	0.000208
Texture	$8.64e^{-06}$	$9.77e^{-06}$	− 0.885	0.376508
Perimeter	$1.56e^{-03}$	$1.35e^{-05}$	115.061	$<2.0e^{-16}$
Area	$-2.31e^{-06}$	$7.88e^{-07}$	− 2.934	0.003479
Smoothness	$1.15e^{-02}$	$4.48e^{-03}$	2.561	0.010706
Compactness	$-4.72e^{-02}$	$2.64e^{-03}$	− 17.85	$<2e^{-16}$
Concavity	$-7.81e^{-03}$	$1.55e^{-03}$	− 5.05	$-5.98e^{-07}$
Concave points	$-6.1e^{-03}$	$4.49e^{-03}$	− 1.359	0.174854
Symmetry	− 1.79$e^{-03}$	$1.79e^{-03}$	0.999	0.318224
Fractal dimension	$-3.18e^{-02}$	$1.32e^{-02}$	2.412	0.016197
$R^{2}=0.9994$	F statistic: $9.184e^{4}$	p-value: $<2.2e^{-16}$	AIC = − 6383.895	BIC = − 6331.769

Table 4 shows the estimation of the linear regression coefficient The model performance indicators include $R^{2}=0.9994$, F statistic: $9.184e^{4}$ and a p-value of less than 0.05. The information criteria values are AIC = − 6383.895 and BIC = − 6331.769. These results collectively provide insights into the effectiveness and significance of the linear regression model in capturing the relationship between the predictor variables and the response variable.

To check the existence of multicollinearity in the data, two methods are used. First, the correlation matrix of all explanatory variables^41^, Table 5 shows the correlation matrix. It is seen that there are correlations greater than 0.8 between Perimeter and Area, Texture and Concave Points, and Area and Compactness. Second, Variance Inflation Factors (VIF) values for all variables greater than 5^42^, high VIF values are indicative of a strong correlation between the predictor variables. Variables with high VIF: Perimeter, Area, Compactness, Concave Points. The determined condition number $CN=\sqrt{\lambda _{max}/\lambda _{min}}$ of the data is 166.861. The correlation matrix, VIF and CN indicate the existence of a multicollinearity problem.

**Table 5:** The correlation matrix and VIF.

	Diagnosis	Texture	Perimeter	Area	Smoothness	Compactness	Concavity	Concave points	Symmetry	Fractal dimension
Diagnosis	1	0.415	0.743	0.709	0.359	0.597	0.696	0.777	0.330	− 0.013
Texture	0.415	1	0.330	0.321	− 0.023	0.237	0.302	0.293	0.071	− 0.076
Perimeter	0.743	0.330	1	0.987	0.207	0.557	0.716	0.851	0.183	− 0.261
Area	0.709	0.321	0.987	1	0.177	0.499	0.686	0.823	0.151	− 0.283
Smoothness	0.359	-0.023	0.207	0.177	1	0.659	0.522	0.554	0.558	0.585
Compactness	0.597	0.237	0.557	0.499	0.659	1	0.883	0.831	0.603	0.565
Concavity	0.696	0.302	0.716	0.686	0.522	0.883	1	0.921	0.501	0.337
Concave points	0.777	0.293	0.851	0.823	0.554	0.831	0.921	1	0.462	0.167
Symmetry	0.330	0.071	0.183	0.151	0.558	0.603	0.501	0.462	1	0.480
Fractal Dimension	− 0.013	− 0.076	− 0.261	− 0.283	0.585	0.565	0.337	0.167	0.480	1
VIF	3.075	1.305	80.084	56.925	2.933	14.398	11.257	22.476	1.786	6.406

**Table 6:** The estimation of beta regression model parameters.

Coefficient	Estimate	Std. error	Z. value	Pr($>|{{\textbf {Z}}}|$)
Intercept	$$-3.290 e^{+00}$$	$$2.323e^{-02}$$	− 141.651	$$<2 e^{-16***}$$
Diagnosis	$$8.826e^{-03}$$	$$2.221e^{-03}$$	3.975	$$7.05e^{-05***}$$
Texture	$-1.668e^{-04}$	$1.663e^{-04}$	− 1.003	0.316
perimeter	$$2.086e^{-02}$$	$$2.277e^{-0.4}$$	91.609	$$<2e^{-16***}$$
Area	$$-5.877e^{-04}$$	$$1.269e^{-05}$$	− 46.308	$$<2e^{-16***}$$
Smoothness	$$4.202e^{-01}$$	$$7.823e^{-02}$$	5.372	$$7.79e^{-08***}$$
Compactness	$$-6.441e^{-01}$$	$$4.465e^{-02}$$	− 14.425	$$<2e^{-16***}$$
Concavity	$-3.893e^{-02}$	$2.631e^{-02}$	− 1.480	0.139
Concave points	$$-3.445e^{-01}$$	$$7.450e^{-02}$$	− 4.624	$$3.77e^{-06***}$$
Symmetry	− 3.807$e^{-02}$	$3.101e^{-02}$	− 1.228	0.220
Fractal Dimension	$-3.492e^{-01}$	$2.269e^{-01}$	− 1.539	0.124

The highest level of significance is represented by three asterisks (***). If a coefficient has three asterisks, it is statistically significant at the 0.001 significance level. This indicates very strong evidence that the predictor variable has a meaningful impact on the outcome.

Table 6 indicates that several variables (diagnosis, perimeter, area, smoothness, and compactness) have a significant impact on the response variable, while others (texture, concavity, symmetry, and fractal dimension) do not show statistical significance in this analysis. These results provide insights into the relationship between the predictor variables and the response variable in the context of breast cancer data.

**Table 7:** Parameters estimates of GAM regression model.

Coefficient	Estimate	Std. error	Z. value	Pr($>|{{\textbf {Z}}}|$)
Intercept	17.9267965	0.3360811	53.341	$$<2 e^{-16***}$$
Diagnosis	− 0.0958888	0.0318557	− 3.010	0.00273**
Texture	0.0020654	0.0024139	0.856	0.39258
Perimeter	− 0.1742846	0.0032533	− 53.571	$$<2e^{-16***}$$
Area	0.0069018	0.0001733	39.834	$$<2e^{-16***}$$
Smoothness	− 6.4092415	1.1690322	− 5.483	$$6.36e^{-08***}$$
Compactness	4.9832383	0.6318014	7.887	$$1.64e^{-14***}$$
Concavity	0.0339120	0.3652766	0.093	0.92606
Concave points	5.2893625	1.0207720	5.182	$$3.08e^{-07***}$$
Symmetry	0.8430608	0.4545891	1.855	0.06419
Fractal Dimension	11.7227031	3.3034146	3.549	0.00042

The highest level of significance is represented by three asterisks (***). If a coefficient has three asterisks, it is statistically significant at the 0.001 significance level. This indicates very strong evidence that the predictor variable has a meaningful impact on the outcome.

Table 7 views the estimation of GAM parameters and the most influential variables on the response variable. The variables that increase breast cancer according to this data are perimeter, area, smoothness, compactness, and concave points.

**Table 8:** Deviance residuals for different models.

Model	Minimum	1Q	Median	3Q	Maximum
Beta	− 6.4337	− 0.3614	0.0764	0.4294	6.4568
GAM	− 0.170466	0.009906	0.002981	0.014128	0.0243243

Table 8 introduces the residual deviance for the beta and GAM regression model, the deviance residual for the beta regression model ranges from (− 6.4337 to 6.4568), whereas the deviance residuals for the GAM model takes values from (− 0.1704 to 0.243243) which means GAM model has residuals less than beta regression model, this emphasizes that the differences between observed value and estimated value in GAM model are less than these differences in beta model, So the GAM model fits data in a best way from beta regression.

**Table 9:** Parameters estimates of GAM beta regression model.

Coefficient	Estimate	Std. error	Z. value	Pr($>|{{\textbf {Z}}}|$)
Intercept	− 3.290	$$2.346e^{-02}$$	− 140.275	$$< 2e^{-16***}$$
Diagnosis	$$8.826 e^{-03}$$	$$2.242 e^{-03}$$	3.936	$$8.28 e^{-05***}$$
Texture	$-1.668 e^{-04}$	$1.679e^{-04}$	− 0.994	0.320
Perimeter	$$2.086 e^{-02}$$	$$2.299e^{-04}$$	90.720	$$<2e^{-16***}$$
Area	$$5.877 e^{-04}$$	$$1.282 e^{-05}$$	− 45.858	$$<2e^{-16***}$$
smoothness	$$4.202 e^{-01}$$	$$7.900 e^{-02}$$	5.320	$$1.04 e^{-07***}$$
Compactness	$$-6.441 e^{-01}$$	$$4.509 e^{-02}$$	− 14.285	$$<2e^{-16***}$$
Concavity	$-3.893 e^{-02}$	$2.657e^{-02}$	− 1.465	0.143
Concave points	$$-3.445 e^{-01}$$	$$7.523 e^{-02}$$	− 4.579	$$4.68 e^{-06***}$$
Symmetry	$-3.807 e^{-02}$	$3.131 e^{-02}$	− 1.216	0.224
Fractal Dimension	$-3.492 e^{-01}$	$2.291 e^{-01}$	− 1.524	0.127

The highest level of significance is represented by three asterisks (***). If a coefficient has three asterisks, it is statistically significant at the 0.001 significance level. This indicates very strong evidence that the predictor variable has a meaningful impact on the outcome.

Table 9 displays the results of GAM beta regression. It indicates the variables that significantly impact the response variable. Diagnosis, perimeter, area, smoothness, compactness, and concave points have a significant impact on the response variable. Specifically, diagnosis, perimeter, area, and smoothness have positively affected the response variable, implying that these variables increased the risk of breast cancer in the analyzed dataset the breast cancer for patients based on this data. In contrast, compactness and concave points have negatively affected the response variable. They have a decreased risk of breast cancer for patients based on this data. However, the variables texture, Concavity, Symmetry, and Fractal dimension do not show significance. They have small effects on the response variable and are not associated with a significant change in breast cancer risk in this dataset.

**Table 10:** Parameters estimates of ridge regression model.

Coefficient	Estimate (SC)	Estimate	Std. error (SC)	t. value (SC)	Pr($>|{{\textbf {t}}}|$)
Intercept	0.0020	− 70.1006	4.3847	− 15.9876	$$< 2e^{-16***}$$
Diagnosis	0.0005	0.0057	0.0015	3.7369	$$0.0002^ {***}$$
Texture	0.0000	− 0.0009	0.0010	− 0.8858	0.3761
Perimeter	0.0016	0.9021	0.0078	115.1642	$$<2e^{-16***}$$
Area	0.0000	-0.0194	0.0066	− 2.9370	0.0035$$^{ **}$$
Smoothness	0.0115	0.0038	0.0015	2.5631	0.0106 $^{ *}$
Compactness	− 0.0471	− 0.0593	0.0033	− 17.8664	$$<2e^{-16***}$$
Concavity	-0.0078	− 0.0148	0.0029	− 5.0549	$$<2e^{-16***}$$
Concave points	− 0.0061	− 0.0056	0.0041	− 1.3597	0.1745
Symmetry	0.0018	0.0012	0.0012	0.9999	0.3178
Fractal dimension	0.0318	0.0053	0.0022	2.4139	0.0161$^ *$

The highest level of significance is represented by three asterisks (***). If a coefficient has three asterisks, it is statistically significant at the 0.001 significance level. This indicates very strong evidence that the predictor variable has a meaningful impact on the outcome.

Table 10 shows the results of ridge regression, The parameter estimates for the Ridge Regression Model include the estimate of the standard deviation of the error term (SC). It indicates the variables of diagnosis, perimeter, area, smoothness, compactness, concavity, and fractal dimension have a significant impact on the response variable. on the other hand, perimeter has a relatively large impact with an estimate of 0.9021, whereas texture has a very small impact with an estimate of − 0.0009. The variables diagnosis, perimeter, smoothness, and fractal dimension have a positive impact on the response variable. This suggests that an increase in these variables is associated with an increased risk of breast cancer as indicated by the data. In contrast, area, compactness, and concavity have negatively affected the response variable, these variables decreased the breast cancer based on the dataset. However, the variables texture, concave points, and symmetry are not significant in breast cancer risk in this dataset.

**Table 11:** Parameters estimates of beta ridge regression Model.

Coefficient	Estimate (SC)	Estimate	Std. error(SC)	t. value (SC)	Pr($>|{{\textbf {t}}}|$)
Intercept	3.3396	2341.2788	73.3757	31.9081	$$< 2e^{-16***}$$
Diagnosis	0.0053	0.0616	0.0257	2.3985	0.0168 ${*}$
Texture	− 0.0001	− 0.0132	0.0167	− 0.7889	0.4305
Perimeter	0.0216	12.5396	0.1311	95.6586	$$<2e^{-16***}$$
Area	− 0.0006	− 5.3384	0.1105	− 48.3026	$$< 2e^{-16***}$$
Smoothness	0.3929	0.1317	0.0251	5.2493	$$< 2e^{-16***}$$
Compactness	− 0.6751	− 0.8497	0.0556	− 15.2870	$$<2e^{-16***}$$
Concavity	− 0.0179	− 0.0339	0.0491	− 0.6899	0.4905
Concave points	0.3384	− 0.3129	0.0694	− 4.5059	$$< 2e^{-16***}$$
Symmetry	-0.0304	− 0.0199	0.0196	− 1.0144	0.3108
Fractal dimension	− 0.1782	− 0.0300	0.0371	− 0.8086	0.4191

The highest level of significance is represented by three asterisks (***). If a coefficient has three asterisks, it is statistically significant at the 0.001 significance level. This indicates very strong evidence that the predictor variable has a meaningful impact on the outcome.

Table 11 shows the results of beta ridge regression. It indicates the variables of diagnosis, perimeter, area, smoothness, compactness, and concave points have a significant impact on the response variable. on the other hand, perimeter has a relatively large impact with an estimate of 12.5396, whereas texture has a very small impact with an estimate of − 0.0132. The variables diagnosis, perimeter, and smoothness, have a positive impact on the response variable. This suggests that an increase in these variables is associated with an increased risk of breast cancer. In contrast, area, compactness, and concave point have negatively affected the response variable, these variables decreased the risk of breast cancer. However, the variables texture, concavity p, symmetry, and fractal dimension are not significant in breast cancer risk.

**Table 12:** AIC and BIC for different models.

Model	AIC	BIC
GAM	− 4579.654	− 4527.528
Beta	− 5572.542	− 5520.416
GAM. Beta regression	− 5572.435	− 5520.308
Ridge regression	− 8002.647	− 4349.54
Beta. regression	− 4796.358	− 1143.252

According to the model selection criterion, as seen in Table 12, the best model fit is the model that has the lowest value of this criterion; hence the Ridge regression model fits data in the best way for AIC and the Beta regression is the best model for BIC.

**Figure 1:**
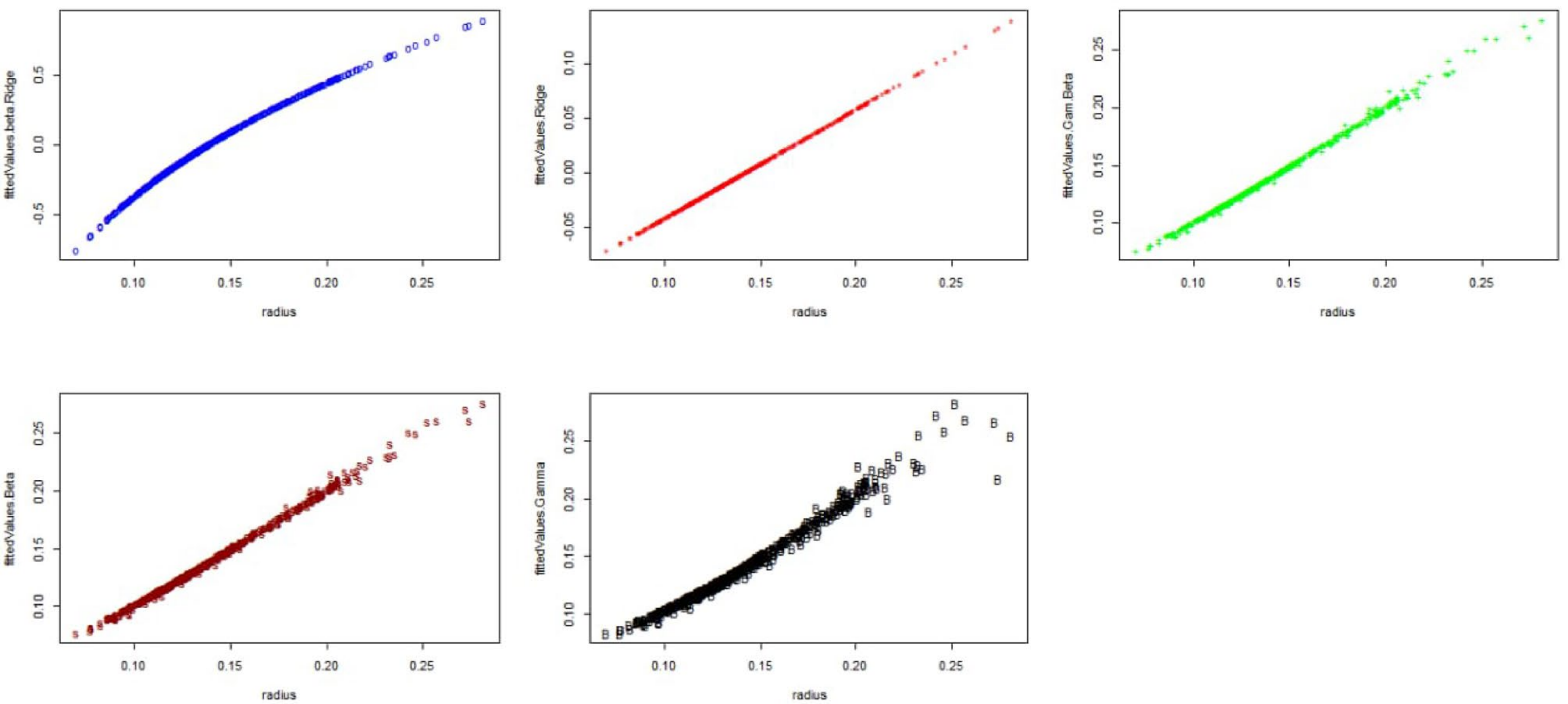
The fitted values of estimated models.

Figure 1, illustrate the estimations of the values for the GAM, Beta, GAM Beta, Ridge, and Beta Ridge models. These figures demonstrate that an increase in one variable’s size corresponds to an increase in another variable, providing evidence that these models effectively capture the relationships between the variables and the radius.

## Monte Carlo simulation study

In this section, we conduct a Monte Carlo simulation experiment to evaluate the performance of our proposed regression models across various conditions. The models under examination include the beta regression model, GAM regression model, GAM Beta regression model, Ridge regression model, and Beta Ridge regression model.

To generate synthetic data for our simulation, we utilized multivariate normal (mvrnorm) and beta distributions using the mvrnorm and rbeta functions, respectively. Specifically, we generated four predictor variables following multivariate normal distribution with a mean vector of zeros and a covariance matrix constructed using a correlation matrix and a diagonal scaling matrix (D). To ensure the stability and reliability of our results, we repeated the simulation 1000 times. We set the true mean parameter for the beta distribution to $\mu$ = 3, with a dispersion parameter of $\phi$ = 15. Given our focus on examining the effect of multicollinearity under different conditions, we varied the degree of correlation (rho) across $\rho = (0.70, 0.80, 0.90)$, and the number of constants (k) across (0, 0.01, 0.10). These parameters allow us to assess the impact of multicollinearity on model performance across a range of scenarios, providing valuable insights into the robustness and applicability of our regression models.

The simulated Akaike information criterion (AIC) and Bayesian information criterion (BIC) introduced by^43^ are criteria for judging the performance of models as follows:

$$\begin{aligned} \text {AIC}=-2 \ln (L_{fit})+2k,\qquad \& \text {BIC}=-2 \ln (L_{fit})+2k \ln (n), \end{aligned}$$

where $\ln (L_{fit})$ is the log-likelihood of whatever model was fitted, *k* is the number of parameters estimated, and *n* is the number of observations^44^. All the computations are performed using the R Programming Language.

### Results and discussion

The results from the Monte Carlo simulations for different n are presented in Tables 13,  14,  15, 16, 17, 18, 19, 20 and 21 respectively. From these tables, the factors affecting the performance of the estimators are the degree of correlation $\rho$, the number of sample sizes *n* and the constant of ridge values *k*. Generally, as the sample size increases, it is expected that both AIC and BIC values will decrease, reflecting the improved fit of the model due to the inclusion of more data points. This decrease is not indicative of a negative relationship between AIC and BIC but rather a reflection of their individual responses to increased sample size. AIC penalizes model complexity to a lesser extent than BIC, which is why they may decrease at different rates as sample size grows. This trend indicates an improvement in the efficiency of all models with larger sample sizes. For all sample sizes, the degrees of correlation, and the constant of ridge values, the Ridge model has the lowest AIC values, indicating a better fit compared to other models, following the Beta and GAM-Beta models have the lowest AIC values, suggesting better fit for larger datasets. Introducing a ridge constant (0.01, 0.10) marginally affects the AIC and BIC values for the Ridge and Beta Ridge models, indicating the sensitivity of these models to regularization strength. on the other hand, for all sample sizes, the Beta and GAM-Beta models have the lowest BIC values, suggesting they provide the best fit for larger sample sizes. The Beta Ridge model has the highest BIC, and AIC values across all sample sizes, indicating a relatively poor fit compared to other models.

**Table 13:** Simulation study results for $\rho =0.7, k=0$.

	AIC				BIC			
	n = 25	n = 50	n = 100	n = 200	n = 25	n = 50	n = 100	n = 200
GAM	− 49.72285	− 104.2512	− 214.7783	− 436.356	− 42.4096	− 92.77904	− 199.1473	-416.5661
Beta	− 50.32005	− 105.138	− 216.0175	− 438.3696	− 43.0068	− 93.66586	− 200.3865	− 418.5797
GAM Beta	− 49.97377	− 104.9769	− 215.9398	− 438.3313	− 42.66051	− 93.50475	− 200.3088	− 418.5414
Ridge	− 122.009	− 244.4311	− 490.1855	− 982.4439	− 36.6616	− 41.18191	− 19.24785	90.41285
Beta Ridge	− 18.11415	− 36.95824	− 75.13192	− 152.8547	67.23325	166.291	395.8058	920.0021

**Table 14:** Simulation study results for $\rho =0.7, k=0.01$.

	AIC				BIC			
	n = 25	n = 50	n = 100	n = 200	n = 25	n = 50	n = 100	n = 200
GAM	− 49.72285	− 104.2512	− 214.7783	− 436.356	− 42.4096	− 92.77904	− 199.1473	− 416.5661
Beta	− 50.32005	− 105.138	− 216.0175	− 438.3696	− 43.0068	− 93.66586	− 200.3865	− 418.5797
GAM Beta	− 49.97377	− 104.9769	− 215.9398	− 438.3313	− 42.66051	− 93.50475	− 200.3088	− 418.5414
Ridge	− 122.2386	− 244.644	− 490.3901	− 982.6441	− 37.03563	− 41.60312	− 19.72371	89.87665
Beta Ridge	− 18.34377	− 37.17109	− 75.33633	− 153.0548	66.8592	165.8698	395.33	919.4659

**Table 15:** Simulation study results for $\rho =0.7, k=0.1$.

	AIC				BIC			
	n = 25	n = 50	n = 100	n = 200	n = 25	n = 50	n = 100	n = 200
GAM	− 49.72285	− 104.2512	− 214.7783	− 436.356	− 42.4096	− 92.77904	− 199.1473	− 416.5661
Beta	− 50.32005	− 105.138	− 216.0175	− 438.3696	− 43.0068	− 93.66586	− 200.3865	− 418.5797
GAM Beta	-49.97377	− 104.9769	− 215.9398	− 438.3313	− 42.66051	− 93.50475	− 200.3088	− 418.5414
Ridge	− 123.3825	− 245.8039	− 491.5689	− 983.8135	− 39.07423	− 44.12146	− 22.71453	86.43907
Beta Ridge	− 19.48874	− 38.32966	− 76.50984	− 154.2226	64.81954	163.3527	392.3445	916.03

**Table 16:** Simulation study results for $\rho =0.8, k=0$.

	AIC				BIC			
	n = 25	n = 50	n = 100	n = 200	n = 25	n = 50	n = 100	n = 200
GAM	− 49.72285	− 104.2512	− 214.7783	− 436.356	− 42.4096	− 92.77904	− 199.1473	− 416.5661
Beta	− 50.32005	− 105.138	− 216.0175	− 438.3696	− 43.0068	− 93.66586	− 200.3865	− 418.5797
GAM Beta	− 49.97377	− 104.9769	− 215.9398	− 438.3313	− 42.66051	− 93.50475	− 200.3088	− 418.5414
Ridge	-122.009	-244.4311	-490.1855	-982.4439	− 36.6616	− 41.18191	− 19.24785	90.41285
Beta Ridge	− 18.11415	− 36.95824	− 75.13192	− 152.8547	67.23325	166.291	395.8058	920.0021

**Table 17:** Simulation study results for $\rho =0.8, k=0.01$.

	AIC				BIC			
	n = 25	n = 50	n = 100	n = 200	n = 25	n = 50	n = 100	n = 200
GAM	− 49.72285	− 104.2512	− 214.7783	− 436.356	− 42.4096	− 92.77904	− 199.1473	− 416.5661
Beta	− 50.32005	− 105.138	− 216.0175	− 438.3696	− 43.0068	− 93.66586	− 200.3865	− 418.5797
GAM Beta	− 49.97377	− 104.9769	− 215.9398	− 438.3313	− 42.66051	− 93.50475	− 200.3088	− 418.5414
Ridge	− 122.3387	− 244.7383	− 490.4813	− 982.7333	− 37.20194	− 41.79316	− 19.93968	89.63332
Beta Ridge	− 18.44389	− 37.26533	− 75.42742	− 153.144	66.69284	165.6798	395.1142	919.2226

**Table 18:** Simulation study results for $\rho =0.8, k=0.1$.

	AIC				BIC			
	n = 25	n = 50	n = 100	n = 200	n = 25	n = 50	n = 100	n = 200
GAM	− 49.72285	− 104.2512	− 214.7783	− 436.356	− 42.4096	− 92.77904	− 199.1473	− 416.5661
Beta	− 50.32005	− 105.138	− 216.0175	− 438.3696	− 43.0068	− 93.66586	− 200.3865	− 418.5797
GAM Beta	− 49.97377	− 104.9769	− 215.9398	− 438.3313	− 42.66051	− 93.50475	− 200.3088	− 418.5414
Ridg	− 123.6609	− 246.1107	− 491.9022	− 984.15	− 39.65438	− 44.90452	− 23.69549	85.28556
Beta Ridg	− 19.76874	− 38.63677	− 76.83921	− 154.5574	64.23779	162.5695	391.3675	914.8781

**Table 19:** Simulation study results for $\rho =0.9, k=0$.

	AIC				BIC			
	n = 25	n = 50	n = 100	n = 200	n = 25	n = 50	n = 100	n = 200
GAM	− 49.72285	− 104.2512	− 214.7783	− 436.356	− 42.4096	− 92.77904	− 199.1473	− 416.5661
Beta	− 50.32005	− 105.138	− 216.0175	− 438.3696	− 43.0068	− 93.66586	− 200.3865	− 418.5797
GAM Beta	− 49.97377	− 104.9769	− 215.9398	− 438.3313	− 42.66051	− 93.50475	− 200.3088	− 418.5414
Ridge	− 122.009	− 244.4311	− 490.1855	− 982.4439	− 36.6616	− 41.18191	− 19.24785	90.41285
Beta Ridge	− 18.11415	− 36.95824	− 75.13192	− 152.8547	67.23325	166.291	395.8058	920.0021

**Table 20:** Simulation study results for $\rho =0.9, k=0.01$.

	AIC				BIC			
	n = 25	n = 50	n = 100	n = 200	n = 25	n = 50	n = 100	n = 200
GAM	− 49.72285	− 104.2512	− 214.7783	− 436.356	− 42.4096	− 92.77904	− 199.1473	− 416.5661
Beta	− 50.32005	− 105.138	− 216.0175	− 438.3696	− 43.0068	− 93.66586	− 200.3865	− 418.5797
GAM Beta	− 49.97377	− 104.9769	− 215.9398	− 438.3313	− 42.66051	− 93.50475	− 200.3088	− 418.5414
Ridge	− 122.597	− 244.99	− 490.7287	− 982.9766	− 37.64199	− 42.31262	− 20.53779	88.95524
Beta Ridge	− 18.70215	− 37.5169	− 75.67426	− 153.3871	66.25281	165.1605	394.5166	918.5447

**Table 21:** Simulation study results for $\rho =0.9, k=0.1$.

	AIC				BIC			
	n = 25	n = 50	n = 100	n = 200	n = 25	n = 50	n = 100	n = 200
GAM	− 49.72285	− 104.2512	− 214.7783	− 436.356	− 42.4096	− 92.77904	− 199.1473	− 416.5661
Beta	− 50.32005	− 105.138	− 216.0175	− 438.3696	− 43.0068	− 93.66586	− 200.3865	− 418.5797
GAM Beta	− 49.97377	− 104.9769	− 215.9398	− 438.3313	− 42.66051	− 93.50475	− 200.3088	− 418.5414
Ridge	− 124.0771	− 246.5816	− 492.4416	− 984.6949	− 40.65164	− 46.31017	− 25.52098	83.10588
Beta Ridge	− 20.18901	− 39.10716	− 77.36955	− 155.0993	63.23644	161.1643	389.5511	912.7015

**Figure 2:**
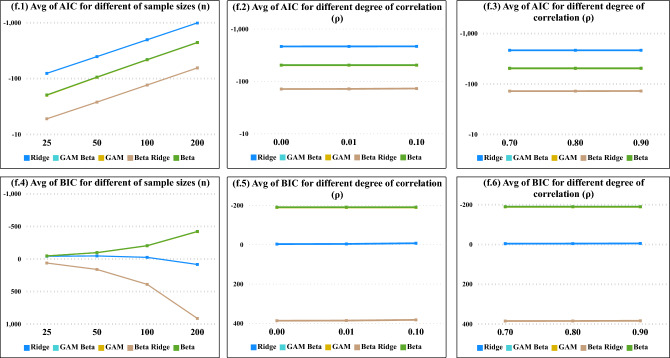
Average Values of AIC and BIC the degree of correlation ($\rho$), the number of sample sizes (*n*) and , and the constant of ridge values (*k*) for all models.

**Figure 3:**
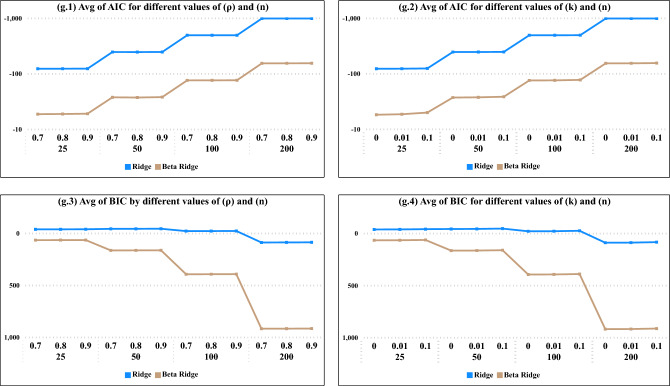
Average Values of AIC and BIC the degree of correlation ($\rho$) and the constant of ridge values (*k*) for BRR, RR models.

From Fig. 2, we found that as sample sizes increased the average values of AIC decreased, Moreover, the average values of both AIC and BIC demonstrate a decreasing pattern with the increase in sample sizes, specifically evident in the beta regression model, as illustrated in Figs.1 and 2. In Fig. 3, as the degree of correlation $(\rho )$ increases from 0.70 to 0.90, and as the ridge constant (*k*) in all sample sizes, the performance of the Ridge and Beta Ridge models shows a significant fluctuation in both AIC and BIC values, underscoring the sensitivity of these models to changes in correlation and regularization strength.

In Fig. 3, across all values of $(\rho )$ and (*k*), the optimal model fit was consistently observed at a sample size of 200. Specifically, for AIC, the Ridge model demonstrated the best fit, while according to BIC, the Beta model also emerged as the top-performing model.

**Figure 4:**
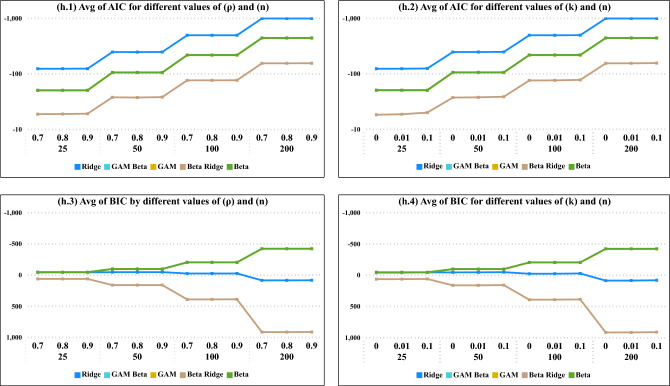
Average Values of AIC and BIC the degree of correlation ($\rho$) and the constant of ridge values (*k*) for all models.

In Fig. 4, as sample sizes increase, depicted in (h.1) for varying degrees of correlation ($\rho$) and in (h.2) for constant ridge values, there is a notable decrease in the average AIC values. Ridge regression consistently emerges as the best model in terms of AIC. Conversely, when assessing the average BIC values, the optimal model is identified as beta ridge regression.

## Conclusions

In this paper, we meticulously tailored a suite of models, including Generalized Additive Models (GAM), Beta regression, GAM Beta regression, Ridge regression, and Beta Ridge regression, to the intricacies of breast cancer data. Our analysis underscored a preference for the Akaike Information Criterion (AIC) in GAMs, attributed to its accommodation of the models’ complexity and flexibility, essential for capturing the multifaceted nature of the data [10].

A thorough simulation study was conducted to empirically validate our models across varying sample sizes and correlation coefficients, enhancing the robustness of our findings. In analyzing data from 569 breast cancer patients, we discerned key independent variables that significantly influence breast cancer risk. The comparative analysis revealed that the Beta regression model outperformed others based on the Bayesian Information Criterion (BIC), while the Ridge regression model showed superiority according to the Akaike Information Criterion (AIC). These results mirror those obtained from our simulation study, indicating that the selection between Ridge and Beta regression models may depend on the preferred information criterion, especially in smaller sample sizes. However, as sample sizes increase, both models consistently demonstrate suitability across both AIC and BIC metrics. It is crucial to acknowledge that these conclusions are drawn within the confines of our study’s dataset and simulation parameters, necessitating caution when extrapolating to other contexts.

Our investigation rigorously assessed a suite of low-dimensional regression models, including Generalized Additive Models (GAM), Beta regression, GAM Beta regression, Ridge regression, and Beta Ridge regression. While acknowledging the extensive research on high-dimensional regression models^27,28^, our study is distinctively focused on low-dimensional contexts. Applied to authentic breast cancer data, the performance of these models was meticulously evaluated against a simulation study, ensuring a robust examination within the dataset’s dimensional constraints.

## Data Availability

The data in this study was obtained from an open online repository from https://www.kaggle.com/code/gpreda/breast-cancer-prediction-from-cytopathology-data.
